# Ozonization of Air in the Sick Room

**Published:** 1873-11

**Authors:** 


Ozonization of Air in the Sick Room.—Dr. Lender (*Deutsch Klinik*, No. 19, 1873), proposes an easy means of carrying out the above object. He mentions the use of a powder composed of peroxide of manganese, permanganate of potash, and oxalic acid, which has the property of giving out, in contact with water, an abundant quantity of ozone. For a chamber of middling size he uses about two tablespoonfuls of the powder, over which he pours from one to one and a half tablespoonfuls of water every two hours. In this way the quantity of ozone produced is exactly what is wanted; the presence of a larger quantity in the air would occasion a cough. All kinds of metals, except gold and platina, must be removed from the room, on account of the oxidizing effects of ozone.

Treatment of Burns and Scalds.—Dr. de Breyne highly recommends the following treatment in *L'Union Pharmaceutique*: Hydrate of lime, (newly precipitated), forty-five grains; glycerine, five ounces; chloric ether, forty-five drops. It makes up a transparent, colorless liquid, with an agreeable odor, and an alkaline reaction, according to the dose of hydrate of lime. It calms the pain, and prevents or abates inflammation.

